# Ultrafast infrared spectroscopy reveals water-mediated coherent dynamics in an enzyme active site†
†Electronic supplementary information (ESI) available. See DOI: 10.1039/c4sc02752c



**DOI:** 10.1039/c4sc02752c

**Published:** 2014-10-22

**Authors:** Katrin Adamczyk, Niall Simpson, Gregory M. Greetham, Andrea Gumiero, Martin A. Walsh, Michael Towrie, Anthony W. Parker, Neil T. Hunt

**Affiliations:** a Department of Physics , University of Strathclyde , SUPA , 107 Rottenrow East , Glasgow , G4 0NG , UK . Email: neil.hunt@strath.ac.uk; b Central Laser Facility , Research Complex at Harwell, STFC Rutherford Appleton Laboratory, Harwell Oxford , Didcot, Oxon , OX11 0QX , UK; c Diamond Light Source , Diamond House, Harwell Science and Innovation Campus , Didcot, Oxfordshire , OX11 0DE , UK

## Abstract

Understanding the impact of fast dynamics upon the chemical processes occurring within the active sites of proteins and enzymes is a key challenge that continues to attract significant interest, though direct experimental insight in the solution phase remains sparse. Similar gaps in our knowledge exist in understanding the role played by water, either as a solvent or as a structural/dynamic component of the active site. In order to investigate further the potential biological roles of water, we have employed ultrafast multidimensional infrared spectroscopy experiments that directly probe the structural and vibrational dynamics of NO bound to the ferric haem of the catalase enzyme from *Corynebacterium glutamicum* in both H_2_O and D_2_O. Despite catalases having what is believed to be a solvent-inaccessible active site, an isotopic dependence of the spectral diffusion and vibrational lifetime parameters of the NO stretching vibration are observed, indicating that water molecules interact directly with the haem ligand. Furthermore, IR pump–probe data feature oscillations originating from the preparation of a coherent superposition of low-frequency vibrational modes in the active site of catalase that are coupled to the haem ligand stretching vibration. Comparisons with an exemplar of the closely-related peroxidase enzyme family shows that they too exhibit solvent-dependent active-site dynamics, supporting the presence of interactions between the haem ligand and water molecules in the active sites of both catalases and peroxidases that may be linked to proton transfer events leading to the formation of the ferryl intermediate Compound I. In addition, a strong, water-mediated, hydrogen bonding structure is suggested to occur in catalase that is not replicated in peroxidase; an observation that may shed light on the origins of the different functions of the two enzymes.

## Abstract 2

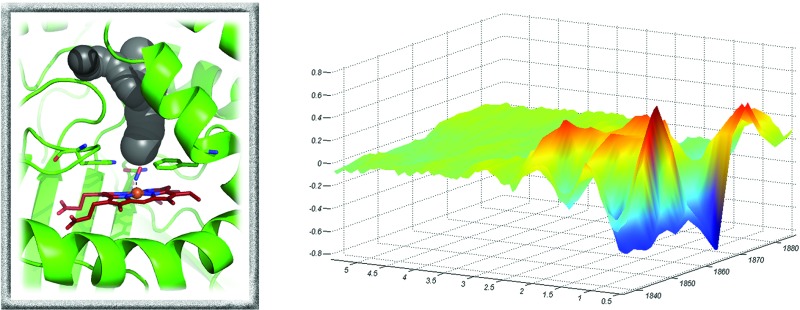
Ultrafast infrared spectroscopy provides insights into the dynamic nature of water in the active sites of catalase and peroxidase enzymes.

## Introduction

Understanding the chemistry that drives enzyme function represents a major challenge. In particular, determining the aspects of biomolecular structure that promote specific reactivity will be central to the future design of synthetic species for novel bio-medical applications. One key question yet to be addressed relates to the degree to which fast structural dynamics influence processes that take place many orders of magnitude more slowly.^1^ While protein dynamics have been widely measured in the nanosecond to millisecond time regimes, the role of ultrafast (picosecond) motions remains elusive.^1–5^

Running parallel with these issues is the behaviour of the solvent during biological processes. A detailed understanding of the dynamic nature of water in enzyme active sites or the potential for mechanistic involvement of structural or mobile water molecules is yet to be achieved, largely due to the technically-challenging nature of the experiments required to measure them. It remains a distinct possibility however that the fast dynamics of proteins are irrevocably linked to those of the solvent on these timescales.^6–9^

Here, we report a series of ultrafast infrared spectroscopy measurements focusing on the structural, vibrational and solvent dynamics present in the active site of the catalase enzyme, in which the mechanistic role of water is a topic of some debate.^10,11^ By drawing on comparisons with similar measurements on the closely-related peroxidases where the situation regarding water involvement is clearer, we seek to shed new light on the influence of both ultrafast dynamics and solvent molecules on the functioning of these important enzymes.

The catalases are haem-based enzymes, common to almost all aerobically-respiring organisms, where they form part of cell defense mechanisms by catalysing the disproportionation of hydrogen peroxide. The process can be summarised as:^12–14^

1 catalase–Fe(iii) + H_2_O_2_ ⇒ O

<svg xmlns="http://www.w3.org/2000/svg" version="1.0" width="16.000000pt" height="16.000000pt" viewBox="0 0 16.000000 16.000000" preserveAspectRatio="xMidYMid meet"><metadata>
Created by potrace 1.16, written by Peter Selinger 2001-2019
</metadata><g transform="translate(1.000000,15.000000) scale(0.005147,-0.005147)" fill="currentColor" stroke="none"><path d="M0 1440 l0 -80 1360 0 1360 0 0 80 0 80 -1360 0 -1360 0 0 -80z M0 960 l0 -80 1360 0 1360 0 0 80 0 80 -1360 0 -1360 0 0 -80z"/></g></svg>

Fe(iv)Por^+^˙ + H_2_O

2 OFe(iv)Por^+^˙ + H_2_O_2_ ⇒ catalase–Fe(iii) + H_2_O + O_2_

**Fig. 1:**
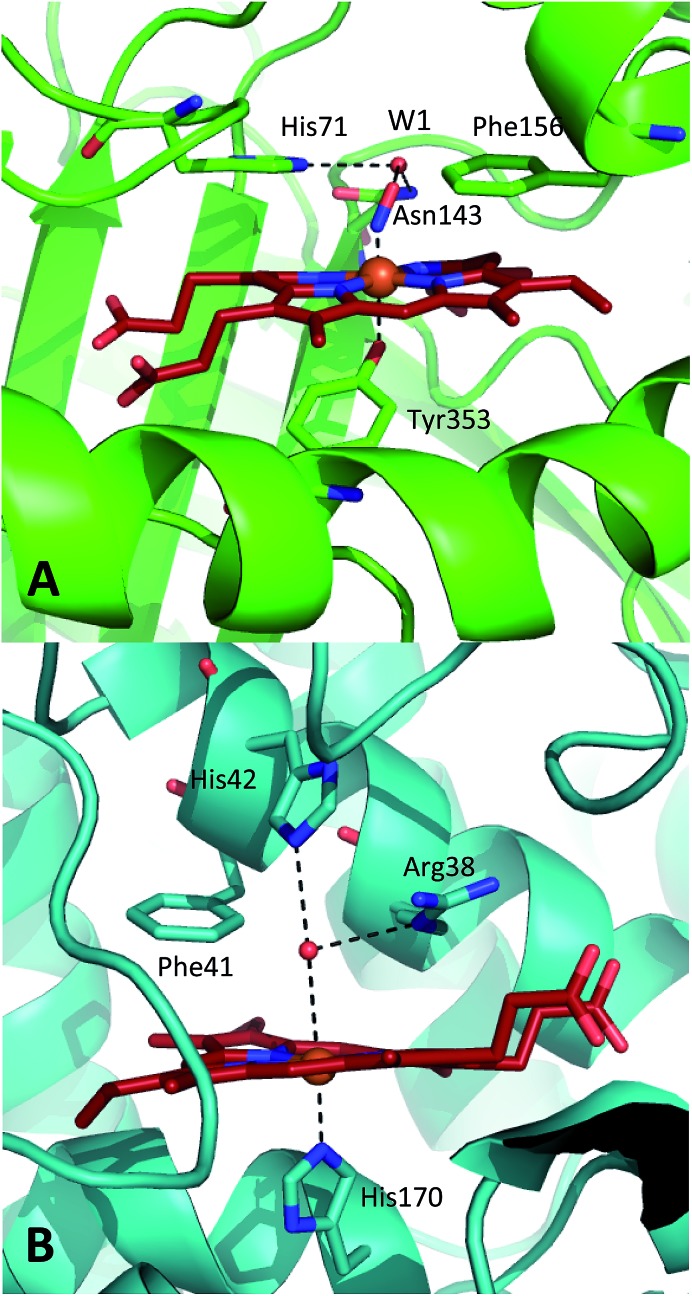
Comparison of the structures of the active sites of (A) nitrosylated catalase from *C. glutamicum* (PDB code: ; 4B7F (ref. 25)) and (B) HRP (pdb: ; 1atj (ref. 39)). Coordination and hydrogen bonds are shown as dashed lines and the main residues discussed in the text are indicated. The haem group is shown as red sticks with water represented by red spheres.

2H_2_O_2_ ⇒ 2H_2_O + O_2_(1)+(2)where OFe(iv)Por^+^˙, referred to as Compound 1 (CpdI), is an oxy-ferryl centre with a radical delocalised on the porphyrin ring. This two-stage representation of the mechanism is widely accepted, but the precise molecular interactions underpinning them remain unclear. The catalases possess a highly conserved active site, featuring a distal pocket that includes histidine and asparagine residues located near the haem centre (Fig. 1A).^15–17^ The distal histidine in particular is widely implicated in the catalase mechanism and mutation studies have shown that this residue is crucial to CpdI formation.^18–20^ Furthermore, EPR measurements indicate that transfer of a proton to the imidazole moiety on the distal histidine (His71, Fig. 1A) accompanies coordination of the first molecule of hydrogen peroxide to the ferric haem, leading to a precursor species *en route* to CpdI.^19^ This intermediate, called Cpd0, has been shown to take the form Fe(iii)–OOH.^21^ Ultimately, further similar proton transfer events are also thought to accompany the reduction of CpdI during reaction (2).^22^

A similar situation exists in the peroxidases (Fig. 1B), where the mechanism also follows step (1) to reach CpdI *via* Cpd0. Subsequent steps differ however in that the peroxidase CpdI undergoes two one-electron reduction steps with the concomitant oxidation of organic substrates bound near the haem rather than the single two-electron step found in the catalases. In the case of peroxidases, conserved histidine (*e.g.* His42 in Horseradish peroxidase, Fig. 1B) and arginine (Arg38) residues have been shown to play important roles in proton transfer and substrate binding steps leading to elements of structural similarity between the two haem pockets.^23^

Important differences arise between these two enzyme types when the role of water in the active site is considered. The catalase haem is deeply buried and accessed only by channels in the protein architecture^24^ and so is widely held to be non-solvent accessible, in stark contrast to the solvent accessible peroxidase haem pocket. This feature has been linked to the difference in function between the enzymes:^11^ It is proposed that the water molecule formed during reaction (1) remains close to the haem in peroxidases, generating a so-called ‘wet’ variant of CpdI whereas the catalase mechanism is thought to be facilitated by the water molecule being efficiently removed from the vicinity of CpdI; the so-called ‘dry’ intermediate.^11^ The concept of such a ‘dry’ intermediate would seem consistent with the high efficiency of the catalase enzyme, which implies rapid transfer of reactants and products to and from the active site. However, models predict that the access channel to the haem site has a similar affinity for both H_2_O and H_2_O_2_. This suggests that the concept of solvent-accessibility may be somewhat more subtle,^24^ for example with channels blocking bulk solvent access but allowing rapid access/escape of small numbers of H_2_O/H_2_O_2_ in certain molecular geometries.

Further indications as to the central role of water molecules in the operation of these enzymes follow from suggestions that the distal histidine group in catalase is insufficiently basic to abstract a proton from hydrogen peroxide without some additional contributing chemical factor arising from within the protein pocket.^10^ This is somewhat contradictory to the identification of this histidine as a primary proton sink during the steps leading to CpdI formation. Moreover, there is evidence that the interaction between the distal histidine and the haem ligand may be mediated by a structurally-conserved water molecule (hereafter W1, see Fig. 1A) rather than being a direct association.^10,25^ Indeed, catalases feature several such conserved water molecules in the haem pocket, of which W1 is of particular interest due to its close proximity to the haem centre.^16,25,26^ These water molecules do not feature strongly in mechanistic studies and it has been suggested that they are expelled during CpdI formation, although crystal structures appear to contradict this and have supported a W1-mediated proton transfer mechanism.^27^ It is thus plausible that the presence and position of water molecule W1 in conjunction with the distal histidine is fundamental to Cpd0 formation and possibly subsequent steps. Inevitably, mutation of the distal pocket will disrupt the local environment such that W1 and the distal histidine may become inseparable in mutation-based studies and thus obscure the precise molecular interpretation of the mechanism.

The involvement of water in the peroxidase mechanism seems clearer. Several structural studies have highlighted the presence of both conserved structural water molecules, similar to W1, and so-called disordered solvent molecules, near the haem.^10,28–32^ Furthermore, crystal structures suggest that distances between the haem ligand and key distal residues are larger than usual for H-bonding partners and molecular dynamics simulations have shown that water molecules are the correct size to bridge these gaps. In turn, inclusion of these water molecules into computational models leads to reductions of the predicted activation energy barriers for important mechanistic steps, such as transfer of a proton from H_2_O_2_ to the distal histidine and the subsequent O–O bond cleavage that leads to CpdI formation.^33,34^ Finally, structural and biochemical evidence exists for the involvement of water molecules in the peroxidase mechanism, for example, ordered, bridging water molecules are found in crystal structures of CpdI,^35^ while kinetic isotope effects point to the formation of Grothuss-like proton wires in ascorbate peroxidase.^32^ Experiments on horseradish peroxidase (HRP) in isotopically-labelled solvent indicate that water plays a part in the reaction to produce CpdI,^36^ though alternative explanations for these data have been proposed.^33^

It is clear that a direct experimental measurement of the chemical and dynamic nature of the active sites of these enzymes, including the behaviour of nearby water molecules, will shed vital new light on some of these important issues. In particular, comparisons between catalase and peroxidase will be instructive in seeking the reasons for their different functionalities. Recently, 2D-IR experiments on ferric catalase revealed a structurally and dynamically constricted active site that is unusual in comparison to other haem proteins.^25^ In conjunction with X-ray crystallography, the relative structural rigidity was ascribed to the presence of a conserved water network surrounding the haem group, one of which (W1) was suggested to interact directly with nitric oxide bound to the ferric haem centre.^25^ To date, direct measurements of equilibrium ultrafast structural or water dynamics are not available for any peroxidase in its biologically-active ferric state. A 2D-IR study of the ferrous carbonmonoxy form of HRP was reported, though no contribution from water to the dynamic measurements was indicated.^37^ Ligand rebinding studies of NO to HRP following UV-visible wavelength photolysis have been carried out.^38^ These were consistent with a solvent-accessible active site.

Here, we report ultrafast 2D-IR and IR pump–probe measurements of the structural dynamics and the vibrational lifetime of NO bound to the ferric haem of catalase and HRP in both H_2_O and D_2_O solvents in order to conclusively determine the nature of the interaction between the haem ligand and the active site architecture. In addition to isotope dependence of the dynamics in both enzymes, these results reveal the presence of coherent motions in the catalase active site that couple to the haem ligand stretching motion and which may be pivotal to the enzymatic mechanism.

## Results

### FTIR spectroscopy

Nitrosylation of ferric catalase in H_2_O (hereafter referred to as ‘aqueous’) solvent led to the observation of a new band in the infrared absorption spectrum, located at 1881.1 cm^–1^, which could be well-represented by a single Gaussian lineshape function with a full width half maximum (FWHM) of 7.8 cm^–1^ (see Fig. S1(A and B) ESI†). This is consistent with previous studies that assigned this peak to the NO stretching vibration (*ν*_NO_) of nitric oxide bound to the iron atom of the haem group.^25^ When a D_2_O (deuterated) solvent was used, neither the frequency nor FWHM of this absorption was observed to change significantly, yielding a frequency of 1881.0 cm^–1^ and a width of 7.3 cm^–1^.

In the case of HRP, similar peaks were observed (Fig. S1(C and D)†), located at 1903.9 cm^–1^ (aqueous) and 1902.7 cm^–1^ (deuterated) with FWHM values of 13.9 and 14.7 cm^–1^ respectively. The results of fitting the *ν*_NO_ bands in each sample to a Gaussian lineshape function are shown in Table 1.

**Table 1:** Results of fitting ultrafast spectroscopy data (see text)

		Catalase		HRP	
		H_2_O	D_2_O	H_2_O	D_2_O
FTIR	Frequency^*a*^ (cm^–1^)	1881.1	1881.0	1903.9	1902.7
	Linewidth (cm^–1^)	7.8 ± 0.06	7.3 ± 0.10	13.9 ± 0.24	14.7 ± 0.20
IR_pump–probe_	Vibrational lifetime (ps)	9.9 ± 0.3	14.9 ± 0.5	14.8 ± 0.3	18.5 ± 0.5
	Oscillation freq. (cm^–1^)	12 ± 3; 36 ± 5		—	
2D-IR	Spectral diffusion (ps)	1.3 ± 0.2	1.7 ± 0.1	3.3 ± 0.6	7.4 ± 1.0

^*a*^Error < ± 0.05 cm^–1^ in all cases.

### IR pump–probe spectroscopy

**Fig. 2:**
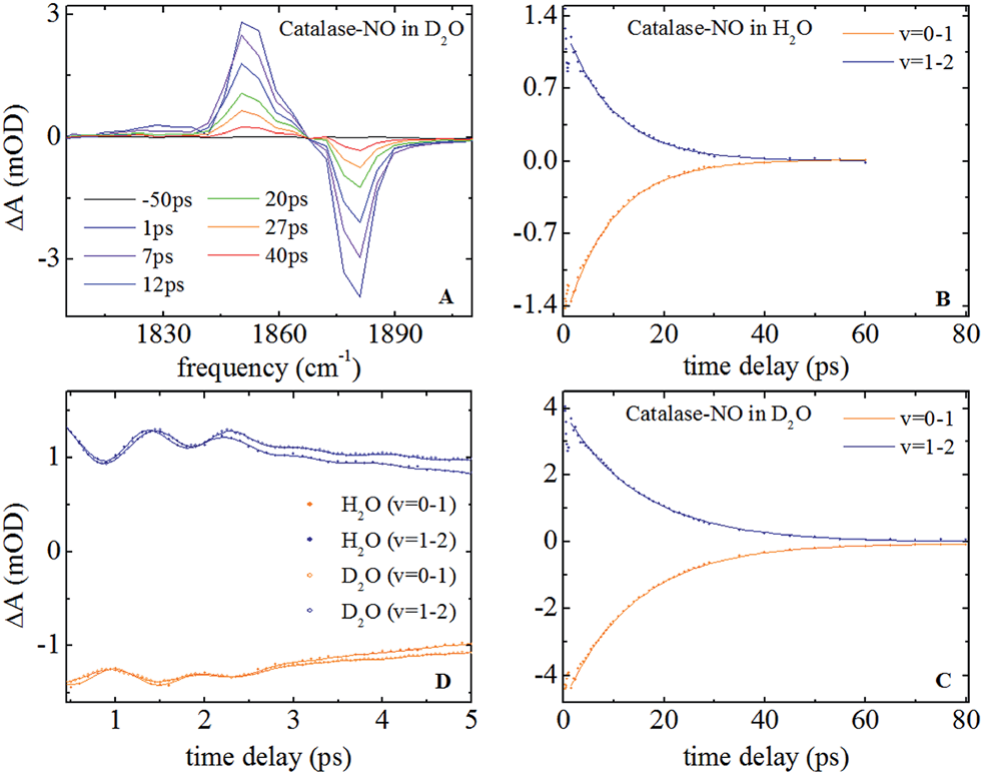
(A) Infrared pump–probe spectra of nitrosylated catalase in D_2_O at a range of pump–probe delay times. (B and C) relaxation dynamics of the *v* = 0–1 and *v* = 1–2 transitions of the *ν*_NO_ mode in H_2_O and D_2_O solvents respectively. The points show experimental data while the solid lines are fits to exponential functions. (D) IR pump–probe data showing oscillations observed in both solvents. The data in D_2_O has been scaled to facilitate direct comparison with that in H_2_O.

Representative IR pump–probe spectra for catalase at a range of pump–probe delay times are shown in Fig. 2A. In both aqueous and deuterated solvents a negative peak was observed at a frequency of 1881 cm^–1^, corresponding to the bleaching and stimulated emission of the *v* = 0–1 transition of the *ν*_NO_ mode. This was accompanied by a positive, transient absorption, feature located at 1850 cm^–1^ assignable to the *v* = 1–2 transition of this mode. Both peaks were observed to decrease in amplitude as a function of pump–probe delay time (Fig. 2B and (C)) in a manner that was well-represented by exponential decay functions with almost identical timescales; representing the vibrational relaxation time of the *v* = 1 level of the *ν*_NO_ mode. The parameters extracted from the fitting process are shown in Table 1. A vibrational lifetime of 14.9 ps was observed in deuterated solvent, which is consistent with previous measurements that reported a value of 16 ps.^25^ The small variation reflects the greater number of data points collected herein. In aqueous solvent, the vibrational relaxation time was observed to reduce to 9.9 ps, indicating that the vibrational relaxation time increases by a factor of 1.5 upon deuteration.

An oscillatory component was also observed in the pump–probe data that was superimposed upon the exponential decay in both solvents at delay times shorter than ∼4 ps (Fig. 2D). Subtraction of the exponential decay due to vibrational relaxation and Fourier Transformation (FT) of the residual oscillations indicated the presence of two component frequencies: 12 ± 3 and 36 ± 5 cm^–1^ (see Fig. S2†). Following subtraction, the residual oscillations were also fit to the sum of exponentially damped sine functions, revealing a damping time constant for the dominant higher frequency component of 1.2 ± 0.1 ps in both solvents. The oscillation was found to be reproducible, though the relatively small magnitude of the feature in comparison to the overall pump–probe signal resulted in the ±3–5 cm^–1^ spread in the frequencies recovered *via* FT from several measurements. As a consequence, no clear dependence of these frequency components upon solvent was observed but a small variation cannot be categorically ruled out.

IR pump–probe data for HRP are shown in Fig. S3.† These show similar spectral features to those of catalase. Once again the vibrational lifetime of the NO stretching vibration was found to vary with solvent; values of 14.8 and 18.5 ps being recovered for aqueous and deuterated samples respectively. The values recovered are summarised in Table 1. No oscillatory features were observed in pump–probe experiments on HRP samples, though it is noted that the lower concentration of HRP and the greater linewidth of the *ν*_NO_ absorption somewhat reduced the signal to noise ratio of the pump–probe data obtained for HRP samples with respect to catalase.

### 2D-IR spectroscopy

**Fig. 3:**
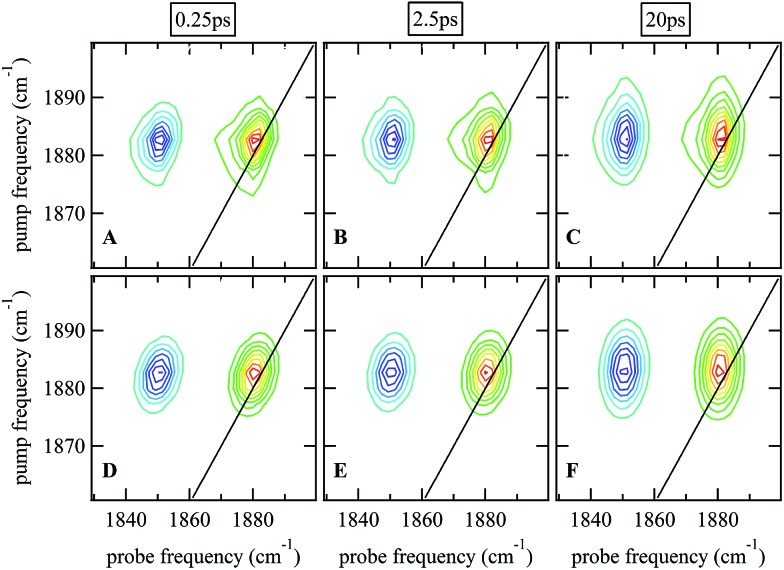
(A–C) 2D-IR spectra of nitrosylated catalase in D_2_O at a range of waiting times. (D–F) Fits of the data in (A–C) to 2D Gaussian lineshape functions, see text.

Ultrafast 2D-IR spectra of the *ν*_NO_ mode of nitrosylated catalase in deuterated solvent are shown in Fig. 3(A–C); the corresponding data for aqueous samples are shown in Fig. S4(A–C).† The data in deuterated solvents are consistent with those reported previously.^25^ The diagonal (*v* = 0–1) transition of the *ν*_NO_ mode is located at 1881 cm^–1^ while the accompanying *v* = 1–2 peak is shifted by 31 cm^–1^ to lower probe frequency. The *ν*_NO_ mode was observed to demonstrate inhomogeneous broadening, as demonstrated by an elongation of the 2D lineshape along the spectrum diagonal at short experimental waiting times (equivalent to a pump–probe delay time). As the waiting time increased, the lineshape became more circular in profile.^25,40–43^ This effect is caused by fluctuations in the electrostatic environment of the ligand leading to small, dynamic changes in the haem ligand stretching frequency.^44,45^ At short waiting times this leads to a diagonal elongation of the 2D-IR peaks because the experimental timescale is short relative to the fluctuation dynamics, causing a correlation of pump and probe (excitation and detection) frequencies in the 2D-IR spectrum. As the waiting time increases to become comparable to the underlying dynamics, this correlation is lost as the ligand samples the full range of environments accessible to it, leading to a circular lineshape. This process is termed spectral diffusion and quantification of the waiting-time-dependent changes in the lineshape provides a measure of the structural dynamics of the haem ligand environment. A range of methods has been established to achieve this quantification.^8,9,43,46–49^

**Fig. 4:**
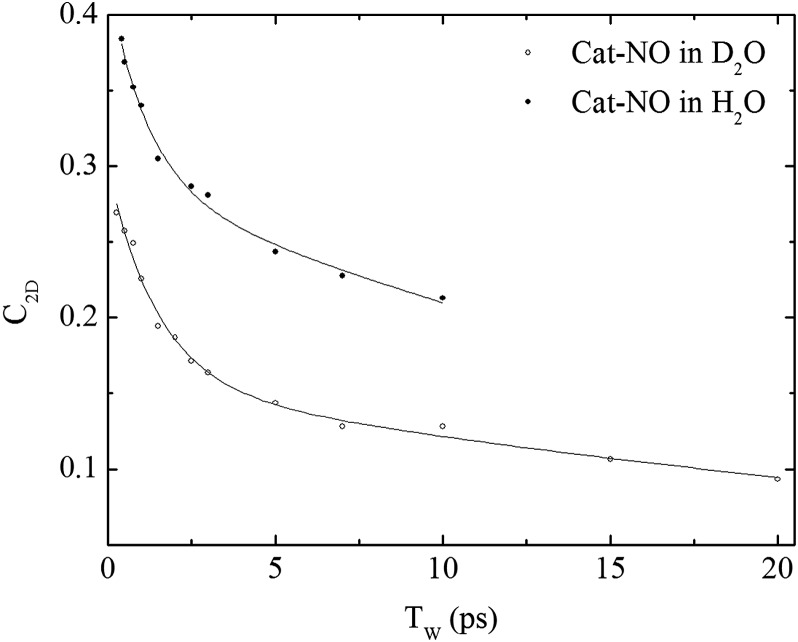
Spectral diffusion of the *ν*_NO_ mode of nitrosylated catalase in D_2_O and H_2_O as a function of waiting time.

The spectral diffusion dynamics for the *ν*_NO_ mode of catalase–NO in both solvents were extracted *via* fitting to a 2D Gaussian function incorporating a parameter, *C*_2D_, which provides a quantification of the correlation between pump and probe frequencies. This method has been reported previously and compared to other approaches.^43^ The results of the spectral fitting for deuterated (aqueous) samples are shown in Fig. 3D–F (Fig. S4(D–F)†) while the dependence of the *C*_2D_ parameter upon the waiting time is shown in Fig. 4 for both solvents.

In a previous study reporting the spectral diffusion dynamics of catalase–NO in deuterated solution, we employed a single exponential function to fit the temporal decay of *C*_2D_ and obtained a value of 3 ps for the spectral diffusion dynamics.^25^ Using a similar approach with the new data reported here returned a value of 2.5 ps, again with a small static offset, which is in close agreement when taking into account the greater number of data points obtained at short waiting times in this study. It was clear from the data for catalase–NO in deuterated solvent (Fig. 4) however that superior agreement between fitting function and data could be obtained using a biexponential function, albeit with a second decay constant that was too long to be well-defined, considering the temporal range of the data obtainable. This approach resulted in a shortening of the picosecond timescale decay recovered and highlights the dependency of the absolute value of the picosecond dynamics upon data points obtained at longer waiting times where the signal to noise ratio is lower due to vibrational relaxation. This was particularly the case for aqueous solutions where the larger spectral density of the solvent in the NO stretching region (Fig. S1†) reduced the signal somewhat in comparison to that in D_2_O. In the case of aqueous solutions, fitting to a biexponential function was found to be possible but the quality of the fit was insensitive to the long timescale parameter. Thus, to reliably compare the short waiting time dynamics in the two solvents, a biexponential function was employed with the longer decay parameter fixed in order to minimise the error at shorter times. This accounts for the fact that longer-timescale processes influence the spectral diffusion in both cases but that these are not accurately recoverable. Using this method, picosecond timescale decays of 1.3 ± 0.2 ps and 1.7 ± 0.1 ps were obtained for aqueous and deuterated samples respectively. Furthermore, fitting the data to single exponential functions over a range of waiting time windows in order to assess the impact of the long-time dynamics revealed a range of values for the two samples of 1.1–1.5 ps (aqueous) and 1.6–1.8 ps (deuterated) leading to the conclusion that, irrespective of the absolute value of the dynamics, which will also be affected by faster processes, a clear isotope dependence is observed in the picosecond window. This can be observed in Fig. 4.

The corresponding 2D-IR data for HRP-NO samples is shown in Fig. S5,† where similar features to those observed for catalase are present. Extraction of the spectral diffusion dynamics in this case resulted in clear single-exponential behaviour with decay timescales of 3.3 and 7.4 ps recovered for aqueous and deuterated HRP samples respectively. In each case a substantial static offset parameter was observed. The values recovered are summarised in Table 1.

## Discussion

### Vibrational lifetimes

The observation of vibrational lifetime parameters for both catalase and HRP that vary with the isotopic composition of the solvent potentially provides important new insights into the chemical nature of the active sites of these enzymes. The strong isotope dependence indicates that exchanging D for H in the solvent facilitates vibrational energy transfer from the NO stretching vibration to other modes within the sample. The identity of these modes is not discernible from this experiment but it is clear that they, or the coupling between them and the NO ligand are influenced by the isotopic identity of the solvent.

Two possible reasons exist for these observations: firstly that the NO ligand is effectively solvated by water, giving rise to ‘water-like’ vibrational relaxation mechanisms that would be influenced by H–D exchange. This would imply that a sufficient number of solvent molecules are present in the vicinity of the ligand as to enable this vibrational relaxation mechanism. Secondly, it is possible that the isotopic exchange of the accessible protons in the protein scaffold leads to a change either in the spectral density of states of the protein or to a specific change in the frequency of the distal histidine side chain vibrations. In either case, deuteration may affect the coupling between the NO stretching vibration and other protein vibrations, giving rise to an isotope dependence of the vibrational lifetime.

In considering these two mechanisms, it is instructive to refer to additional vibrational lifetime measurements carried out on nitrosylated ferric myoglobin (Fig. S6†). Myoglobin is a globular protein, as are catalase and HRP, which displays a direct contact between its distal histidine residue and diatomic haem ligands. As such, it is to be expected that similar H–D exchange effects would be observed in this system if the origin of the effect were simply a generic process arising from protein deuteration. As can be seen in Fig. S6,† this is not the case, which strongly favours interaction with water molecules as being the origin of the isotope dependence of the vibrational lifetime of the NO stretching modes of catalase and HRP.

Variations in vibrational lifetime upon switching from aqueous to deuterated water solvents have been observed in the case of the cyanide ion^50^ as well as cyano^51,52^ and carbonyl^53^ stretching vibrations of organometallic compounds. In each case, a reduction in vibrational lifetime in aqueous solvents relative to deuterated ones was observed. This was universally attributed to the increased spectral overlap of the solute vibration with the combination band of H_2_O located near 2100 cm^–1^ featuring simultaneous excitation of librational and H–O–H bending vibrations. In aqueous media, rapid vibrational relaxation is facilitated by coupling to this broad solvent mode but this is reduced in deuterated solvents when the combination band shifts to ∼1500 cm^–1^.

The observation of a similar isotope dependence of the vibrational lifetime for the *ν*_NO_ mode of Fe–NO in catalase and HRP suggests that an analogous relaxation mechanism operates in the active site of both enzymes. The lower vibrational frequency of the NO stretching mode in comparison to cyano or metallocarbonyl modes means that, rather than overlapping directly with the H_2_O combination band, the ∼1881 cm^–1^ *ν*_NO_ vibration lies exactly halfway between the solvent combination band and the fundamental bending vibration whereas, in D_2_O, the mode lies between the OD stretching vibration and the combination band. The result of this is that the IR spectra of the nitrosylated enzymes (Fig. S1†) show a considerable increase in solvent-related spectral density in the 1900 cm^–1^ region for H_2_O in comparison to D_2_O. This, along with the previously observed correlation between the vibrational relaxation time of the solute and the IR absorption cross section of the solvent,^50^ leads to the expectation of enhanced vibrational relaxation under aqueous conditions, as is observed.

The IR pump–probe data thus suggest that relaxation of the *ν*_NO_ mode occurs *via* coupling to a distinctly water-like local environment for both enzymes.^24^ This is perhaps not surprising for HRP, which is accepted to have a solvent accessible active site, but it is widely suggested that the catalase active site is non-solvent accessible. This data would however seem to imply that sufficient solvent molecules exist in the active site of catalase to facilitate the relaxation mechanism, though their exact dynamic nature cannot be determined from these pump–probe measurements and we must also consider the spectral diffusion data (*vide infra*).

### Coherent oscillations

The detection of oscillatory features in pump–probe signals following the excitation of the haem ligand stretching vibration in catalase points to an unusual situation in the active site of catalases. As such, it is instructive to consider the origins of the effect. As the experiment excites a single oscillator, the NO stretching mode of the Fe–NO moiety, and the IR absorption is well-represented by a single lineshape function, the oscillations are unlikely to arise from quantum beats caused by the coherent excitation of more than one molecular transition by the pump pulse. Similarly, the oscillatory frequencies observed do not match the observed anharmonicity of the NO stretching mode. This suggests that the oscillatory features are most likely to arise from an anharmonic coupling of the haem ligand stretching mode to one or more low frequency vibrational modes of the nearby enzyme structure, leading to the generation of wavepackets on either the ground or excited vibrational states of the NO stretching mode upon excitation, which lead to oscillations in the signal as they evolve.

Once again two main possibilities would seem to exist that account for these observations. Firstly, it is plausible that the NO ligand vibration couples to vibrational modes of the haem group, to which it is coordinatively bound. An alternative explanation is that the distal part of the catalase haem pocket possesses low frequency modes that couple to the haem ligand perhaps *via* H-bonding.

When considering the haem-coupling mechanism, it is noteworthy that experiments employing electronic excitation of the haem moiety have reported the existence of low frequency vibrational modes in the <50 cm^–1^ range.^54,55^ In particular, the haem doming transition is generally found near 40 cm^–1^ and has been suggested to be important in influencing ligand rebinding.^56^ The oscillations reported here however originate from the excitation of a vibrational transition of the ligand rather than the haem itself. In addition, it would seem reasonable that such a generic coupling between haem ligand and haem would also be present more widely across the haemoprotein family. The additional measurements on nitrosylated myoglobin reported in the ESI (Fig. S6†) show no evidence of oscillatory behaviour and no such observations were made in HRP. This suggests that the effect observed in catalase is not generic to either haem-based enzymes or haem proteins and is particularly interesting in that it raises the prospect of a separation between the catalase and the peroxidase families of enzymes. Finally, we are aware of no reports of similar observations in other haem proteins.

The prospect of the oscillations arising from coupling between the NO ligand vibration and low frequency modes mediated by hydrogen bonds to the haem ligand has some precedent in much simpler solvent–solute systems.^57–59^ Coherent oscillations similar to those observed here have been reported for systems featuring strong hydrogen bonding interactions with the excited mode, such as the acetic acid dimer.^60^ Frequencies between 50 and 170 cm^–1^ were detected in this system following excitation of the hydroxyl stretching transition of the carboxylic acid group. These oscillation frequencies were found to be insensitive to H–D exchange and were assigned to bending and stretching modes of the H-bonded dimer, leading to modulation of the intermolecular hydrogen bonds in which the excited mode was directly involved; highlighting the short-range nature of the coupling.

Analogously, a distal H-bonding arrangement in catalase could be the origin of the oscillations.^60^ The NO ligand contacts the enzyme structure through a hydrogen bond to the distal pocket scaffold, though some discussion exists as to whether this occurs to the distal histidine residue side chain^18–20^ or the conserved bound water molecule W1.^25^ While the data herein do not support a link to the distal histidine, a link through either water or the histidine would be a potential source of the oscillations reported. In this event, the vibrational states of the NO stretching mode create a potential surface for the low frequency modes, meaning that a series of vibrational levels of the low frequency modes are associated with each of the states of the NO oscillator.^60^ Excitation of the *ν*_NO_ mode results in the preparation of a coherent superposition of the low-frequency vibrations and the evolution of the wavepacket created is manifest as an oscillation in the pump–probe signal. Alternatively, Raman-like excitation of the low frequency modes associated with the ground state of the NO oscillator could be caused by the pump pulse, with the coupling of these modes to the NO stretching transition causing the oscillation.

The fact that the oscillations in catalase occur at a lower frequency than those in systems such as the acetic acid dimer are not inconsistent with coupling to such a H-bond network. The frequencies observed are too low to implicate a typical H-bond stretching vibration^61,62^ while the lack of a clear isotope-dependence of the oscillations also rules out a specific, local H-bond stretching vibrational mode. However, insensitivity to isotope exchange was also seen for the deformation of H-bonds caused by motion of the acetic acid dimer. The data for catalase are thus more consistent with coupling occurring to a mode possessing a frequency that is not strongly susceptible to a change in mass of the H–D atom, such as a bending or complex deformation-type mode.

More detail regarding the exact mechanism by which the coupling effect is observed in the pump–probe signal can be obtained from the time and frequency-dependence of the oscillations. Fig. S7† shows the temporal evolution of the spectral lineshapes and peak amplitudes of the pump–probe signal obtained for catalase–NO as well as the time-dependence of the anharmonicity of the transition. This data was obtained by fitting a sum of two Gaussian lineshape functions to the IR-pump/IR-probe spectra, examples of which are shown in S7A. The temporal dependencies of the extracted amplitudes, anharmonicity (Fig. S7B†) and centre frequencies (Fig. S7C†) are also shown. Here it can be seen that the signal increases in magnitude in a correlated fashion with an increase in the anharmonic shift of the *v* = 1–2 transition relative to the fundamental. Such behaviour clearly points to a mechanism by which excitation of the low frequency modes influence the vibrational potential of the NO stretching mode.

There are two possible reasons for the observed behaviour, firstly the low frequency modes influence the anharmonicity of the NO stretching vibration, increasing the separation of the *v* = 0–1 and *v* = 1–2 components. This would reduce the overlap of the positive and negative signals, increasing the observed amplitude. Secondly the low frequency mode could influence the transition dipole moment of the transition as well as the anharmonicity. As the observed values of the linewidth and anharmonicity of the *ν*_NO_ mode indicate little overlap of the positive and negative components, this would seem to support the latter case.

Considering the molecular basis for this behaviour, it is important to note that the HOMO of the NO ligand is an antibonding orbital meaning that H-bonding to the ligand would be expected to increase the vibrational frequency. Thus, strong interactions with a nearby H-bonding partner would be expected both to increase the vibrational frequency of the *ν*_NO_ transition and the anharmonicity of the potential through increased coupling. This would be consistent with the data presented in Fig. S7.† Furthermore, altering the electron density within the NO bond would be expected to influence the *ν*_NO_ transition dipole moment.

Examining the oscillatory behaviour as a function of frequency across the *v* = 0–1 and *v* = 1–2 transitions reveals that the phase of the oscillations varies with frequency (Fig. S8†) in a manner consistent with modification of the transition frequencies as described above, though the relative contributions of the 12 and 36 cm^–1^ components were found to change across the lineshape. In particular, the lower frequency component is most prominent in the wings of the peaks. This provides further support for the presence of dynamic processes which modify the potential of the NO stretching transition. It is not possible to definitively separate the effects of wavepacket motion on the ground *versus* excited states of the *ν*_NO_ transition, though the fact that both parts of the pump–probe lineshape are susceptible to the oscillatory behaviour points to contributions from both mechanisms.^57–59^

In light of the pump–probe measurements on myoglobin (and HRP), which showed no oscillatory behaviour and so seem to preclude direct, through bond, coupling to haem motion, the most plausible explanation of the results is that the NO stretching mode is coupled to motions in the distal haem pocket that modulate the H-bonding to the ligand. The vibrational lifetime data suggests that the primary contact is to water molecules, presumably through W1, but the fact that the observed oscillation frequencies are close to what would be expected for the haem doming transition points to the possibility that there is indirect coupling between the NO and the haem mediated not through the Fe–N coordination bond but through the distal pocket, possibly *via* a strong H-bonding network that is only observable in catalase. This would be consistent with crystallographic data indicating a H-bond link from the haem ligand to the propionate group for this enzyme.^25^

### Spectral diffusion

The results of the 2D-IR spectral diffusion measurements reveal the dynamics that underpin the inhomogeneous broadening of the NO stretching vibration and so contribute further to the molecular picture in the active site of these enzymes. In particular, it has been shown previously that local contacts between the protein scaffold and the NO ligand of the ferric haem influence the fast spectral diffusion dynamics.^43^ Similar observations have been made for carbonmonoxy forms of ferrous haem proteins, though these measurements also indicated that the longer timescale dynamics may be influenced by non-local motions.^63^ The main feature of the spectral diffusion measurements reported here is the fast, picosecond decay that dominates the temporal behaviour of *C*_2D_. This, along with the fact that other vibrational dynamics are clearly influenced by local rather than global effects, suggests that these measurements relate to motions in the immediate vicinity of the ligand that are sensitive to H–D exchange of the solvent for both catalase and HRP.

For catalase in deuterated solvents, the spectral diffusion experiments reveal 1.7 ps dynamics whereas, under aqueous conditions, the timescale shortens to 1.3 ps. In addition to the faster processes, the catalase data also indicates a second exponential decay timescale that is less well-defined but also isotope-dependent. Under either aqueous or deuterated conditions, the data for catalase shows that remarkably little amplitude is expected to remain for the *C*_2D_ parameter at times of ∼30 ps.

It is noteworthy that the fast spectral diffusion timescale reported for catalase–NO is close to that of the NO stretching vibration of sodium nitroprusside (SNP) in D_2_O (1.4 ps).^64^ Furthermore, the experiments on SNP also reported an isotope-dependence; the spectral diffusion timescale shortening to 0.8 ps in H_2_O.^64^ The value of 1.3 ps recovered here for aqueous catalase is consistent with this in terms of the effect of isotope exchange, though the timescales are again somewhat longer. One possible implication of this is that the NO ligand located in the catalase pocket is sensitive to dynamics that are similar to those of an Fe–NO unit in D_2_O solution and that the longer timescale dynamics, not observed for SNP, are attributable to the protein environment. In apparent support of this, it has been shown for other protein systems that ‘bulk water-like’ D_2_O shows a characteristic spectral diffusion timescale of 1–2 ps^7–9^ and the dynamics for catalase–NO are not inconsistent with this. Indeed, the water-like vibrational relaxation behaviour of catalase–NO would also seem to point to this conclusion.

An alternative scenario that must be considered however is that the volume of the haem pocket of catalase available to non-structural water molecules may be small. In this case, rather than acting like bulk solvent, each water molecule will be influenced not only by neighbouring water molecules but also by the polar side chains of protein residues. These bulky side chains would be expected to restrict motions such that the observed dynamics would be in closer agreement with those detected for water in constrained systems such as reverse micelles rather than the behaviour of bulk solution.^65^ Measurements on such micellar systems have shown that, for small numbers of water molecules per surfactant head group, the spectral diffusion dynamics of water slow dramatically with respect to the bulk solution. In the smallest micelles studied, which featured around two water molecules per surfactant head group, decay timescales on the order of 10 ps were reported. In addition to this slow behaviour however a very fast component (<2 ps) was also observed to contribute to the dynamics.^65^ Such a scenario is also consistent with the data obtained from catalase.

**Fig. 5:**
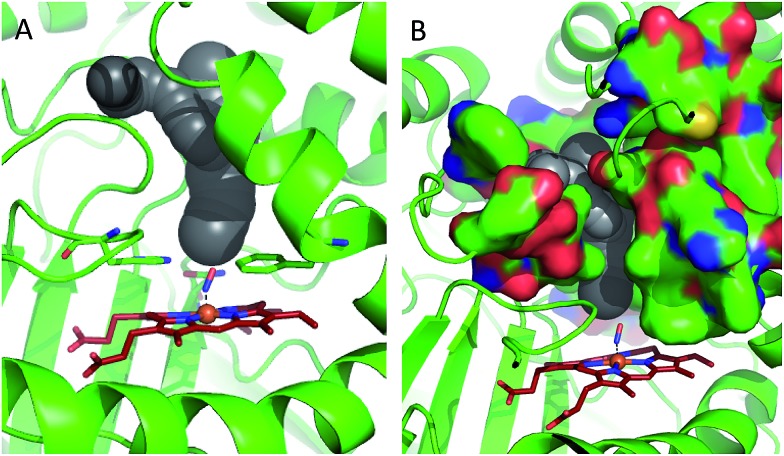
Water accessible cavity (black surface) in NO-bound catalase (pdb: 4b7f).^25^ The cavity volume, estimated with Caver,^66^ corresponds to ∼200 Å^3^. Protein backbone is shown as a green cartoon in A and as green, solvent-excluded surface in B. Images are 30° apart from each other, rotated along the vertical axis. Coordination bonds are shown as dashed lines. Haem is shown as red sticks.

In order to shed some light on the question of whether the bulk water or micellar model is most applicable to the dynamics observed in the catalase haem pocket, the X-ray crystal structure of nitrosylated catalase^25^ was examined to determine the available space within the distal region of the haem pocket that could potentially be filled by non-structural or solvent water.^66^ Using a rolling sphere probe, a cavity with an approximate volume of 200 Å^3^ was identified above the haem, as shown in Fig. 5 and Fig. S9.† According to this model, this void could accommodate approximately 5–6 non-structural water molecules in addition to those identified by the crystal structure itself. These additional water molecules can be seen to reside in a volume that sits directly over the centre of the haem, above the structural water molecule W1, which is likely to be in close contact with the pool (Fig. 5 and S9†). As such, it is entirely feasible that these non-structural species will influence the dynamics of W1 to which the NO is hydrogen bonded.

This analysis would thus seem much more in keeping with the scenario in which the spectral diffusion dynamics of the NO stretching vibration are dominated by water that resides in a constrained environment determined by the structural elements of the haem pocket rather than those of bulk water. In this case, the two decay timescales observed in the spectral diffusion data could both be attributed to water motion within the pocket as in the micellar systems.^65^ This assignment would also be in agreement with the water-dominated vibrational relaxation mechanism and the isotope-dependence of both spectral diffusion timescales.

Another feature of the data that must be considered is that the fast spectral diffusion dynamics observed in catalase are not present in HRP. This invites the possibility that the fast dynamics are related to the origins of the oscillatory signals observed for catalase. Indeed, the timescales for the damping of the oscillations and the fast spectral diffusion dynamics are very similar and it would be expected that the dynamics associated with the loss of coherent behaviour will also be manifest in the spectral diffusion of the NO stretching vibration. As such, it remains a strong possibility that both processes related to the oscillatory behaviour and the dynamics of the confined water pool contribute to the observed fast spectral diffusion behaviour.

The C_2D_ parameter for catalase falls virtually to zero within the experimental duration reported, in clear contrast both to HRP and to other globular proteins.^18–21^ This indicates that many of the processes contributing to inhomogeneous broadening of the *ν*_NO_ transition are complete within this time and that dynamic contributions from the protein scaffold, which normally appear as a pseudo-static parameter in the spectral diffusion data represent a small component of the catalase data. This result has previously been assigned to the presence of a dynamically-constrained active site environment near the haem ligand.^25^ The presence of coherent motions in the pump–probe signal reported here is fully in support of this, suggesting the presence of a local structure that is sufficiently well-defined as to be capable of supporting specific vibrational modes.

The situation for HRP would seem to be rather different to that of catalase. The spectral diffusion data show an isotope-dependent short timescale of 3.3 ps (aqueous) and 7.4 ps (deuterated) alongside a static parameter. The latter is larger in magnitude under aqueous conditions though it is noticeable that the *C*_2D_ parameters recovered in aqueous solvents are larger than in deuterated ones for catalase as well and this may indicate a more global influence of the changing solvent on the protein.

The fast dynamics observed for HRP do not fit with a scenario in which the active site is accessible to bulk-like solvent but nor do they apparently fully match the reverse-micelle-like behaviour of catalase. A similar free-volume calculation carried out on the HRP haem site to that for catalase (Fig. S10†) suggests that the space available for mobile water in HRP is somewhat smaller than catalase (∼60 Å^3^) but still capable of containing a small ‘pool’ of such water molecules.^39^ Perhaps most importantly, the centre of this pool appears to be offset relative to the haem. This suggests that the dynamics of the NO in HRP, which is H-bonded to a nearby structural water molecule that is more distant from the cavity than W1 in catalase, will be dominated by water at the extreme periphery of the water pool. This will lead to the detection of dynamics that, while isotope-dependent because they originate in water motion, are strongly influenced by motions of the protein side chains that are in close proximity to this water. Hence, the fast component is lost and a few picosecond component attributable to restricted water motion remains.

Overall, this suggests that the dynamics experienced by the NO ligand in HRP are again likely to be more consistent with the micellar model of water motion than that of bulk water. In contrast to catalase however, the faster <2 ps component is absent in HRP. As discussed above however, this faster component could also originate in the coherent behaviour present in catalase, not observed in HRP, arising from a well-defined H-bonding structure between residues in the haem pocket.

The assignment of the isotope-dependent 3.3–7.4 ps component to restricted water motion implies that the large static component of the HRP dynamics is attributable to the slower motions of the haem pocket and wider protein. This is well-known for most haem proteins but is absent in catalase.^18–21^ These dynamics indicate that considerably more flexibility is present in the HRP structure, a situation that is consistent with the need to accommodate large and diverse organic substrates in HRP. Such indications of greater flexibility in the HRP system are also in agreement with the observed broader IR absorption linewidth for the NO stretching mode found in HRP than catalase and the faster vibrational relaxation time found for catalase over HRP (Table 1). This implies that the larger distances between the haem ligand and the distal residue side chains observed for HRP lead to a weaker interaction between the NO and nearby water molecules than in catalase and so indicate a firmer, less flexible interaction between the pocket environment and ligand in catalase. Thus, although both catalase and HRP have water-mediated dynamics and similar structures, the active sites offer very different dynamic environments to the haem ligand.

### Mechanistic considerations

Taken together, the IR pump–probe and 2D-IR data indicate that the active sites of catalase and HRP have both similarities and differences. The former arise from the fact that both enzymes have water molecules in close contact with the haem ligands while the latter occur in the dynamic nature of the respective haem pockets. Most notably, the results manifestly support the role of the water molecules in acting as a mediator for proton transfer in the formation of Cpd0 and the subsequent formation of CpdI for both enzymes. This is most likely to be through a W1-like species in both cases though the precise nature of the process would appear to be somewhat different across the two enzymes owing to the well-defined vibrational structure of catalase in comparison to HRP.

It is interesting to consider how the network of coupled vibrational modes and coherent processes observed in the active site of catalase might relate to the reaction coordinate. Such links between fast dynamics and mechanism have been discussed for some systems, most notably for proton transfer steps, but experimental evidence is notoriously difficult to obtain owing to the complexity of enzyme systems.^1,4,5^ Our results measure the presence of at least two vibrational modes that are strongly linked to the haem ligand stretching vibration in catalase. The latter motion is itself identical to the mechanistic step needed to create CpdI, namely cleavage of the inter-heteroatom bond. Together with evidence for coupling between Fe-ligand bond and haem group motion^36–38^ and the apparent influence of the haem doming motion on the H-bond network in catalase, this data provides experimental corroboration for the type of ‘push–pull’ mechanisms for proton transfer suggested from model compound reaction studies, with haem motion and distal H-bonding acting in a concerted manner.^67^

That this motion was not observed in HRP would be consistent with the more flexible pocket needed to accommodate larger substrates than H_2_O_2_ but also suggests that the coherent behaviour may simply assist in the attainment of Cpd0 and CpdI in catalase rather than being vital to it. It is noteworthy that the nature of the catalase active site serves to facilitate vibrational relaxation in comparison to HRP. Such close involvement of the water in redistribution of vibrational energy may be important for the efficiency of the catalytic process by allowing fast relaxation of products or sterically-strained intermediates.

In terms of the established view of the ‘wet’ and ‘dry’ natures of CpdI in peroxidase and catalase respectively, the detection of water-based dynamics for a haem ligand in catalase would seem to be contradictory to this picture. However, it is stressed that the dynamic timescales featured in these measurements lie in the picosecond regime. As such, although the catalase environment is discussed in terms of constrained behaviour, the timescales still provide sufficient mobility that the system will be able to cater for local structural readjustments that facilitate proton transfer. Equally, the susceptibility of active site water to exchange also correlates with the need for efficient macroscopic transfer of water into or away from the active site while maintaining this structured dynamic nature near the haem. Indeed, the latter would seem potentially to promote fast turnover and so represents a refinement of the concept of a ‘dry’ intermediate.

By contrast, taking the assignment of the few picosecond-tens of picosecond dynamic timescale as indicative of restricted water motion for both enzymes, the more flexible HRP environment allows formation of CpdI but with water behaviour that is slightly less dynamically restricted near the haem and so closer to bulk (though by no means fully bulk-like). This may correlate with the macroscopic suggestions of a ‘wet’ intermediate apparently arising from a solvent accessible pocket. Related to these observations, a recently-published structure of cytochrome c peroxidase^68^ indicated that the water molecule equivalent to the W1 considered here is oriented in a manner that would not necessarily favour a H-bond to the haem ligand but was perhaps more indicative of a link to the nearby Arg residue. It was suggested that this may support Arg as the proton carrier required to form CpdI. This observation would also be consistent with work on ascorbate peroxidase^32^ though such arguments do not consider the ultrafast mobility and flexibility of the protein in solution as revealed herein and points to the complementarity of dynamic and crystallographic measurements. It is hoped that these results will inspire further investigation using computational methods but it is apparent that any future model of the catalase mechanism must take into account the clearly-defined structure, dynamics and vibrational modes of this environment.

## Conclusions

Results from studies of the nitrosylated catalase and HRP enzymes in both aqueous (H_2_O) and deuterated (D_2_O) solvents using ultrafast IR pump–probe and 2D-IR studies demonstrate an isotope dependence of the vibrational lifetime and spectral diffusion dynamics of the NO stretching vibration of the ligand. Furthermore the IR pump–probe signal from catalase showed evidence of coherent oscillations arising from coupling of the NO ligand stretching vibration to low frequency vibrational modes. These results are consistent with a direct interaction between the haem ligand and a proposed hydrogen bonding network in the active site of the enzyme mediated by structural water molecules. The observed spectral diffusion dynamics reflect those of confined water although still suggesting that the water molecules are locally mobile within the H-bonded substructure on picosecond timescales. HRP shows evidence of a more flexible protein architecture near the haem along with a slightly more mobile water environment, consistent with the need for binding a wider range of substrates.

Overall, these results provide strong experimental support for hypotheses that the haem ligand is H-bonded to water molecules rather than the distal histidine residue in both catalase and HRP and that this water is central to proton transfer steps leading up to the formation of Compound I. The results also provide experimental evidence for the involvement of coherent, delocalised vibrations in the chemical processes occurring at the active site of some enzymes.

## Materials and methods

For all FTIR, IR pump–probe and 2D-IR experiments, catalase and HRP (both Sigma-Aldrich) were concentrated to the level of ∼1 mM. For experiments carried out under aqueous conditions a phosphate buffer solution (200 mM) was employed at pH7. For deuterated samples, buffer-exchange was performed using pD7 deuterated phosphate buffer solution; care was taken to ensure deuteration of all readily exchangeable protons. MAHMA NONOate was used to nitrosylate the ferric catalase and this was monitored *via* UV-vis spectroscopy as reported previously.^25^ For all IR experiments, the samples were held between two CaF_2_ windows separated by a spacer of 100 μm thickness.

The method for obtaining IR pump–probe and 2D-IR spectra has been described previously. Briefly, 2D-IR spectra were acquired using the FT-2D-IR method described in ref. 25 using a sequence of three mid-infrared (IR) laser pulses arranged in a pump–probe beam geometry (two ‘pump’ pulses and one probe) with parallel relative polarization directions (the effect of molecular rotation on the signals on the timescales studied was found to be negligible).^69,70^ The pulses were generated by a Ti:sapphire laser pumping a white-light seeded optical parametric amplifier (OPA) equipped with difference frequency mixing of the signal and idler. Mid IR pulses with a temporal duration of ∼100 fs; a central frequency of 1900 cm^–1^ with a bandwidth of ∼200 cm^–1^ were employed.

Calculations of the volume of haem cavities as used in the discussion were carried out using Caver^66^ with an inner probe radius of 1.4 Å. All structural images were made using Pymol.^71^
