# Supplementary material for: Ultrafast infrared spectroscopy reveals water-mediated coherent dynamics in an enzyme active site
Source: Chem Sci. 2014 Oct 22;6(1):505–16. doi: 10.1039/c4sc02752c (PMC5588449; doi:10.1039/c4sc02752c)
Supplement: Supplementary file 1 [file SC-006-C4SC02752C-s001.pdf]

Electronic Supplementary Material (ESI) for Chemical Science.
This journal is © The Royal Society of Chemistry 2014

Ultrafast Infrared Spectroscopy Reveals Water-mediated Coherent Dynamics in an Enzyme Active Site

*Katrin Adamczyk,[a†] Niall Simpson,[a] Gregory M. Greetham,[b] Andrea Gumiero,[c‡] Martin A. Walsh,[c] Michael Towrie,[b] Anthony W. Parker,[b] and Neil T. Hunt[a]**

[a]Department of Physics, University of Strathclyde, 107 Rottenrow East, Glasgow, G4 0NG, United Kingdom,

[b]Central Laser Facility, Research Complex at Harwell, Rutherford Appleton Laboratory, Didcot, OX11 0QX, United Kingdom

[c] Diamond Light Source, Harwell Science and Innovation Campus, Didcot, OX11 0DE, United Kingdom

† Present Address: University of Hamburg & MPI for the Structure and Dynamics of Matter, Center for Free Electron Laser Science (CFEL), Luruper Chaussee 149, 22761 Hamburg, Germany

‡ Present Address: Heidelberg University Biochemistry Center (BZH), Im Neuenheimer Feld 328, D-69120 Heidelberg, Germany.
Correspondence email: neil.hunt@strath.ac.uk

Supporting Information:

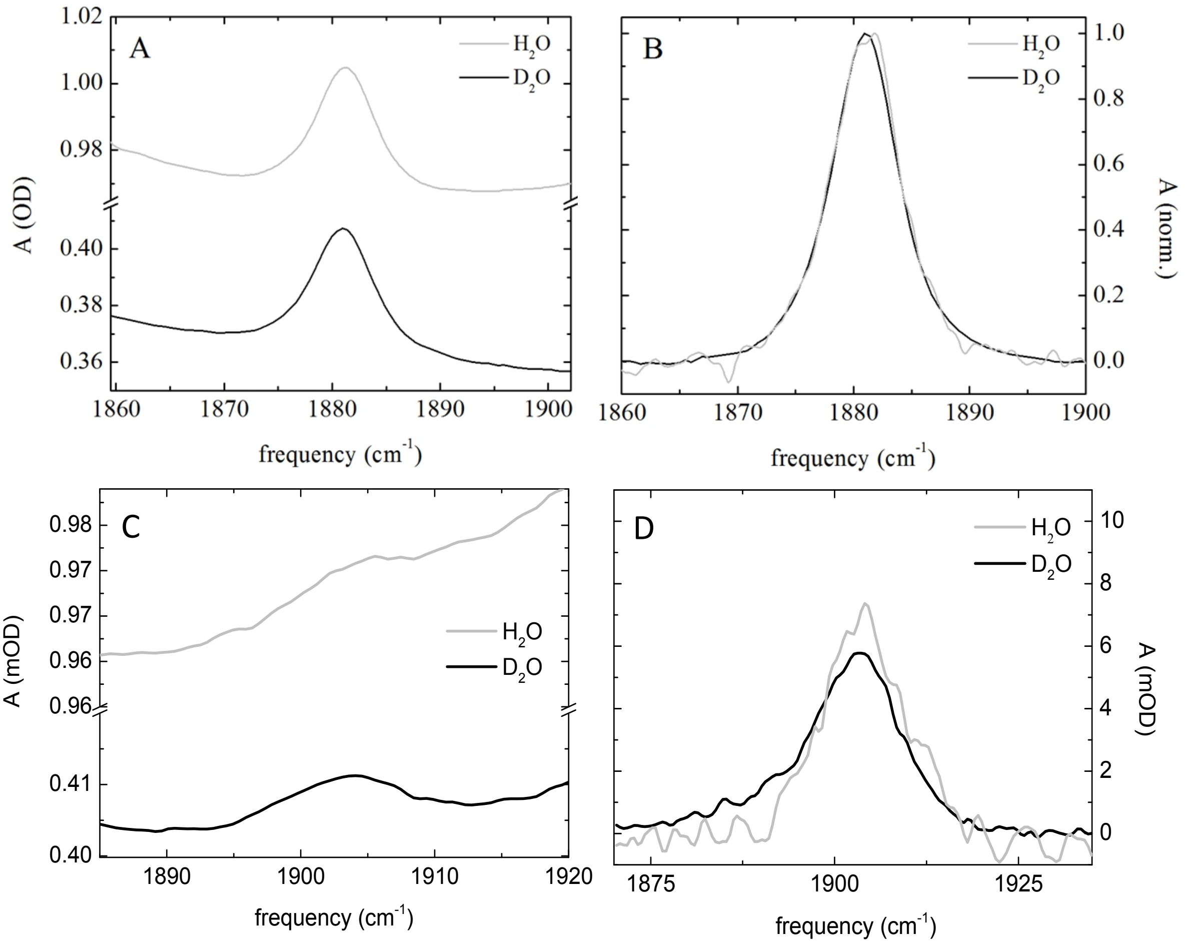

Figure S1: A, FTIR spectra of nitrosylated catalase in H_2O and D_2O-based solvents. The data have not had the solvent response subtracted and it can be observed that H_2O possesses a significantly higher spectral density in this frequency region than D_2O (see main text.) B shows the results of subtracting the solvent response. C, FTIR data for nitrosylated HRP in H_2O and D_2O-based solvents. The figure shows non-solvent subtracted data while D shows the results of removing the solvent contribution. The data in all cases could be fit to Gaussian lineshape functions as described in the main text (Table 1). It is noted that a Voigt lineshape produced a slightly superior agreement with the catalase FTIR data, however, a Gaussian treatment is more consistent both with previous studies and with the lineshapes produced via ultrafast spectroscopy (*vide infra*).

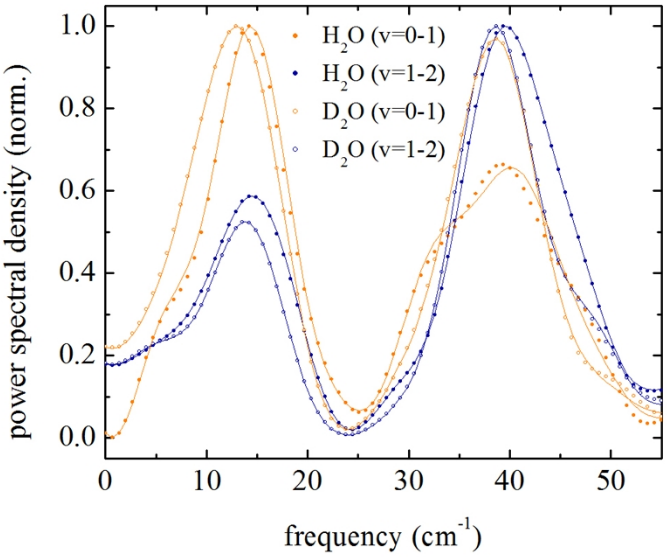

Figure S2: Power spectra obtained following Fourier Transformation of the oscillatory component of the IR pump-probe response of nitrosylated catalase in H_2O and D_2O-based solvents.

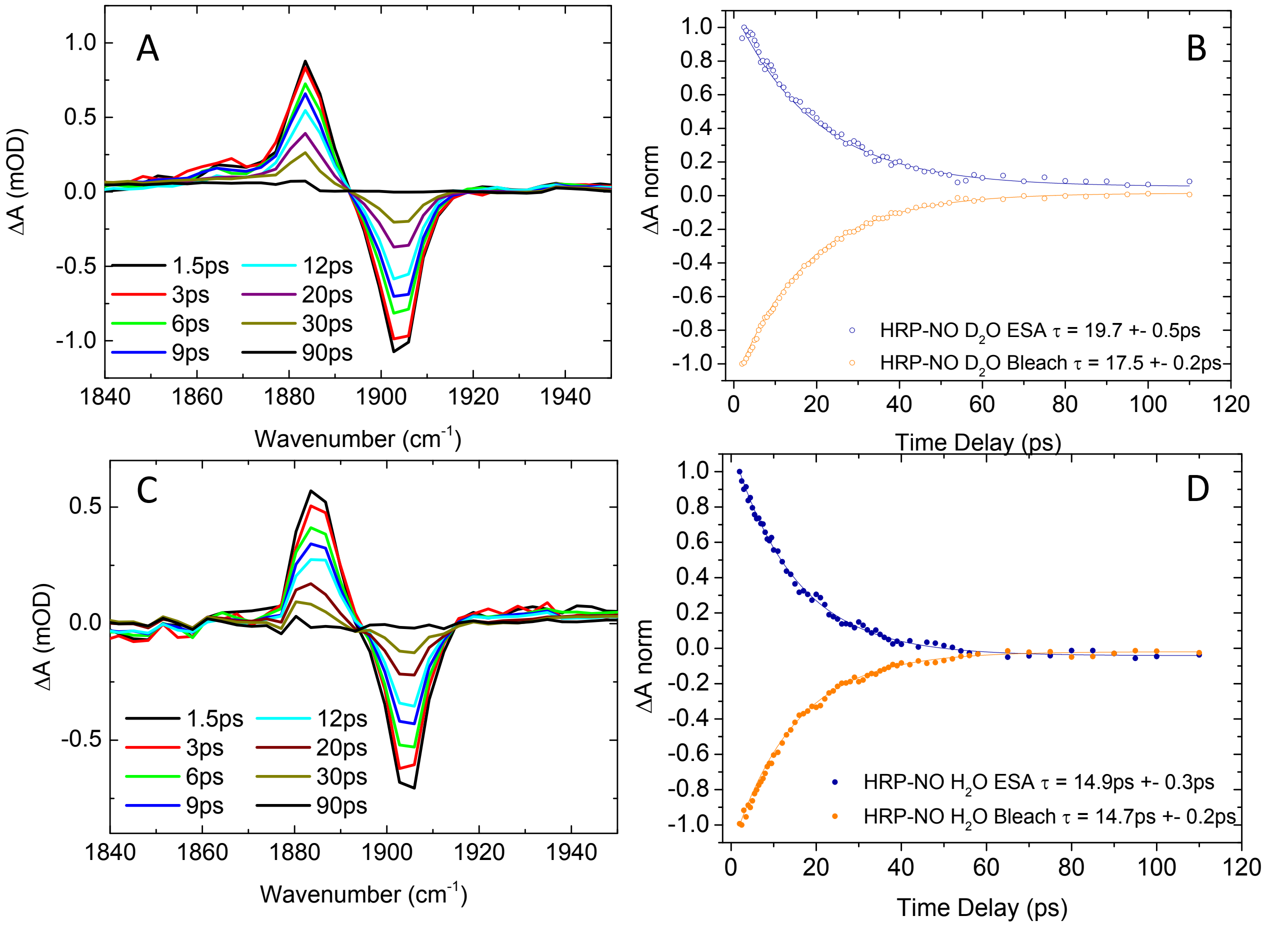

Figure S3: Infrared pump-probe experimental data for HRP-NO showing spectra (A, C) and peak dynamics (B, D) in aqueous (A,B) and deuterated (C,D) solutions respectively.

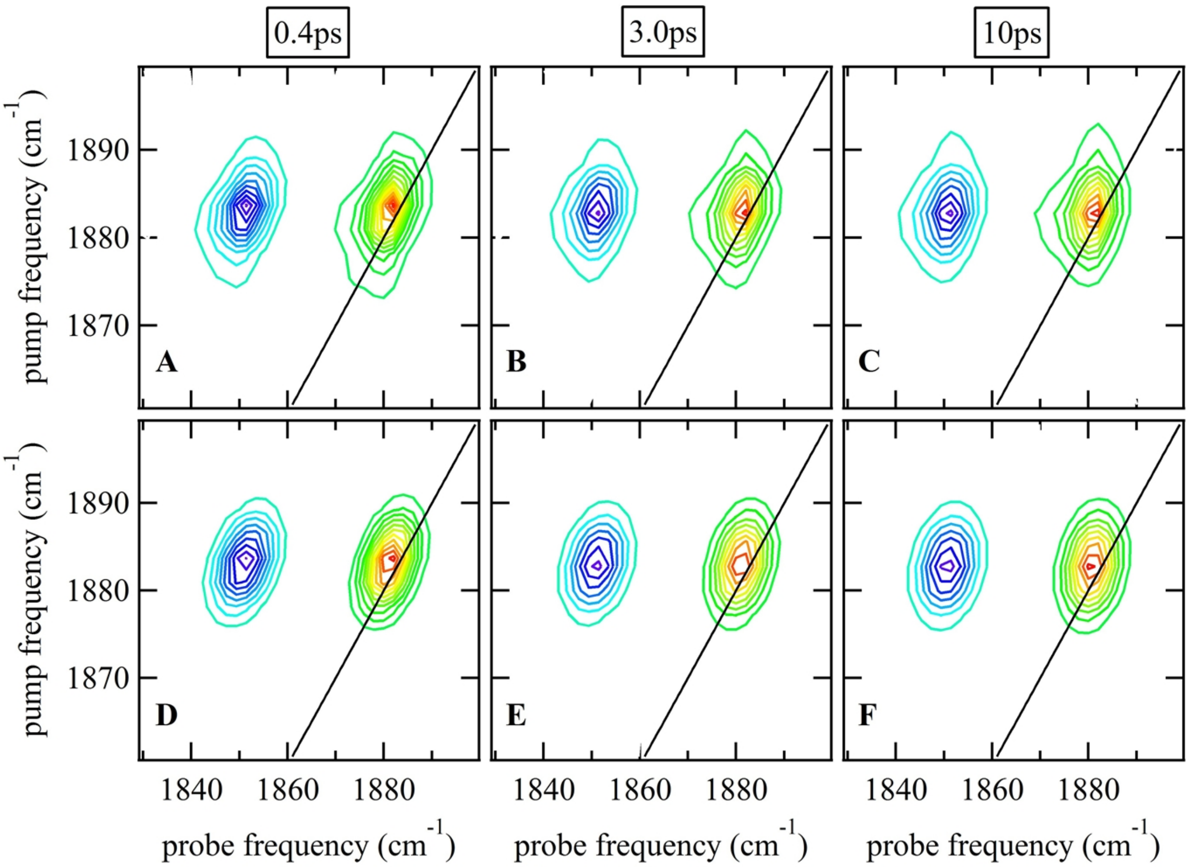

Figure S4: (A-C) 2D-IR spectra of nitrosylated catalase in H_2O at a range of waiting times. (D-F) fits of the data in (A-C) to 2D Gaussian lineshape functions, see main text.

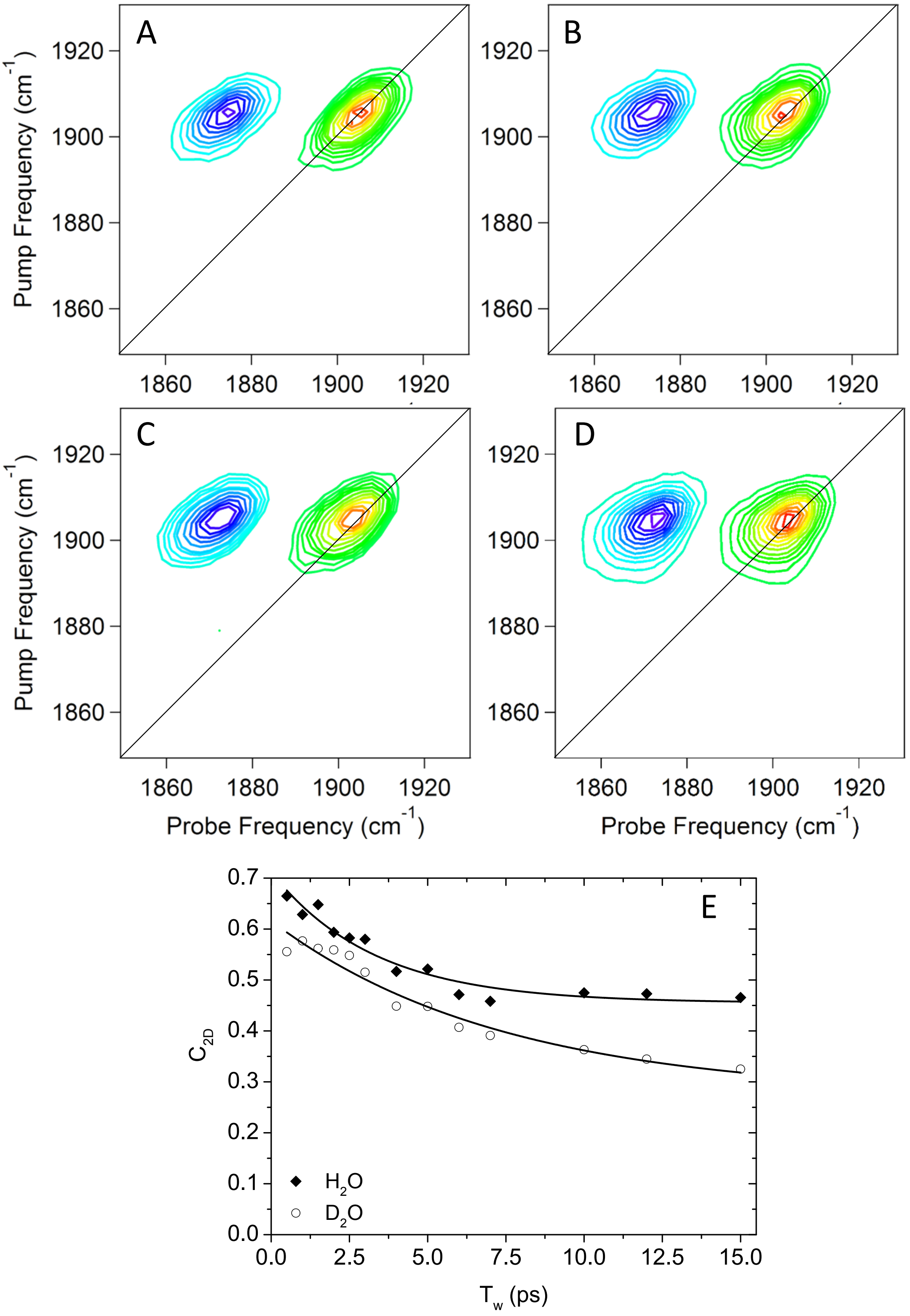

Figure S5: (A-D) 2D-IR spectra of nitrosylated HRP in H_2O (A,B) and D_2O (C,D) at a range of waiting times. (E) Spectral diffusion data extracted from 2D-IR plots for HRP-NO in H_2O and D_2O.

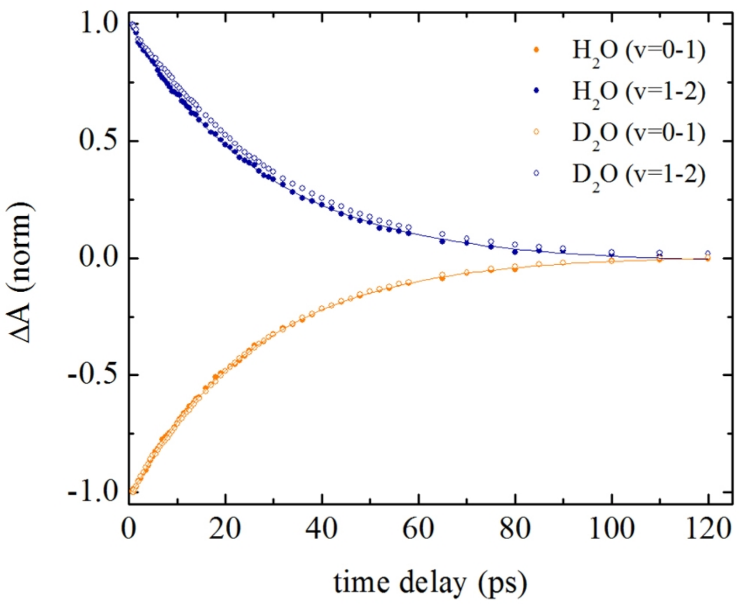

Figure S6: Vibrational relaxation dynamics of the nitric oxide stretching vibration of myoglobin-NO in both aqueous and deuterated solvents. The results of fitting the data to exponential decay functions yield an isotope-independent vibrational relaxation time of 27 ps.

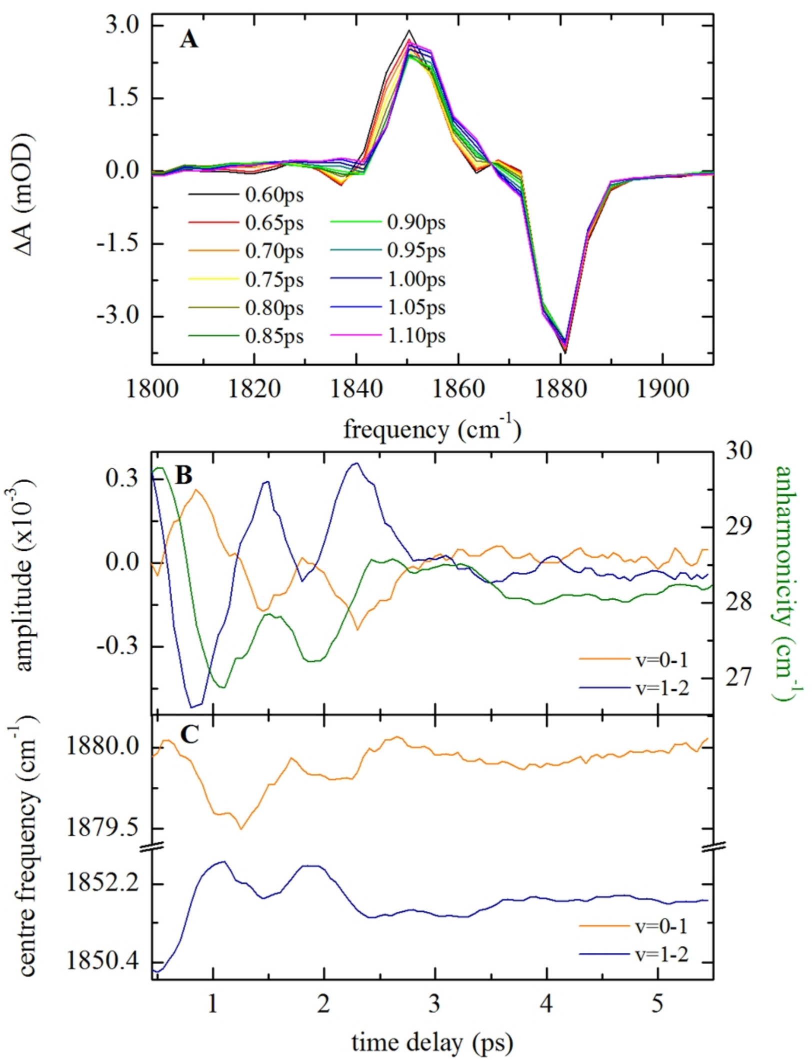

Figure S7: A, The temporal dependence of the lineshapes derived from IR pump-probe measurements on catalase-NO. B, residual oscillatory behaviour of the amplitudes of the v=0-1 (orange) and v=1-2 (blue) transitions following subtraction of effects due to vibrational relaxation. The anharmonic shift of the transition as a function of time is also shown (green, right axis) for comparison. The v=0-1 and v=1-2 transitions have been analysed by fitting a sum of two Gaussian lineshape functions to the IR-pump/IR-probe spectra in A. The extracted amplitude and anharmonicity are plotted in B as a function of time delay. C, temporal dependence of the centre frequency of the v=0-1 and v=1-2 transitions extracted from the fitting procedure described in B.

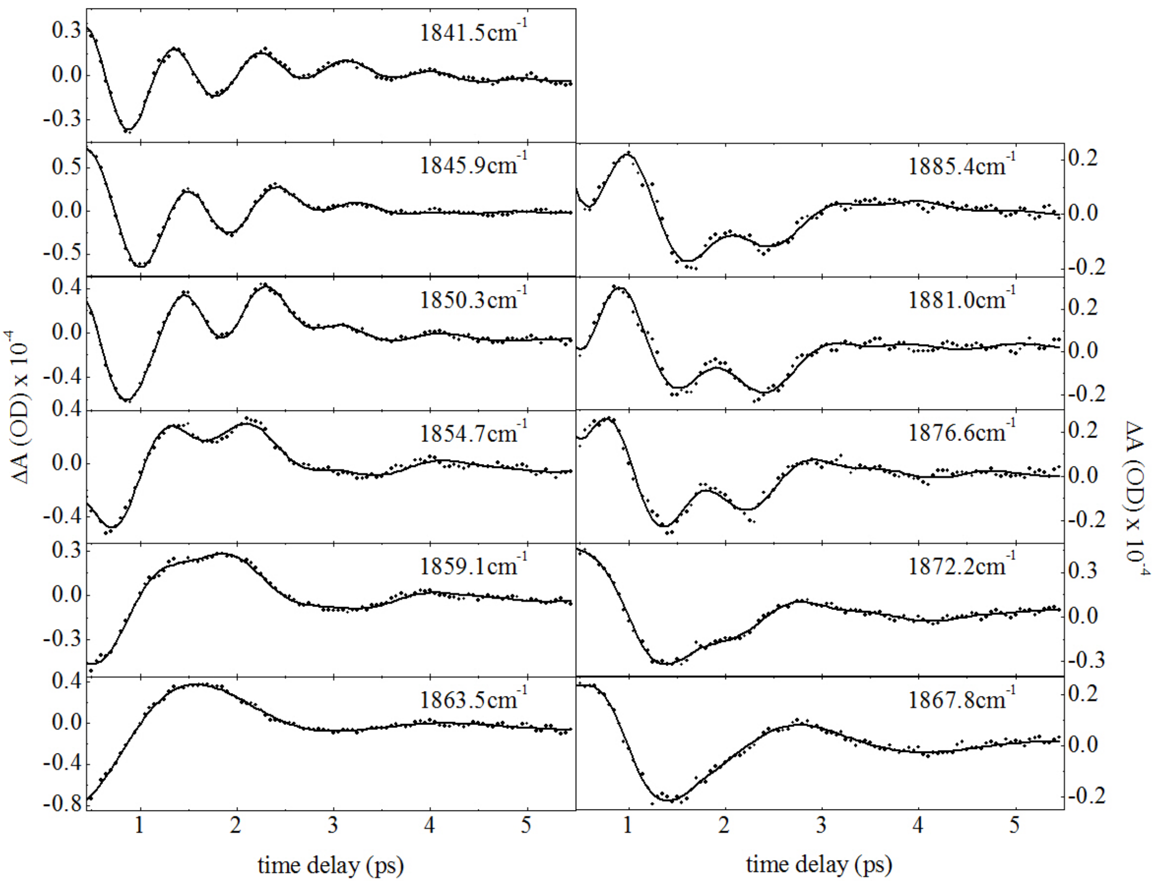

Figure S8: Frequency dependence of the oscillatory behaviour of the catalase-NO IR pump-probe data. The oscillations have been isolated by subtracting the results of fitting a monoexponential decay function to the vibrational relaxation dynamics from the measured transients. The residual oscillatory signals were then Fourier transformed to identify characteristic frequencies. The residual oscillatory components at different frequencies of the excited state absorption and bleach/stimulated emission signals are plotted as dots in the Figure. To analyse the data in more detail the residual oscillations were fit to the sum of exponentially damped sine functions; the results are included as lines.

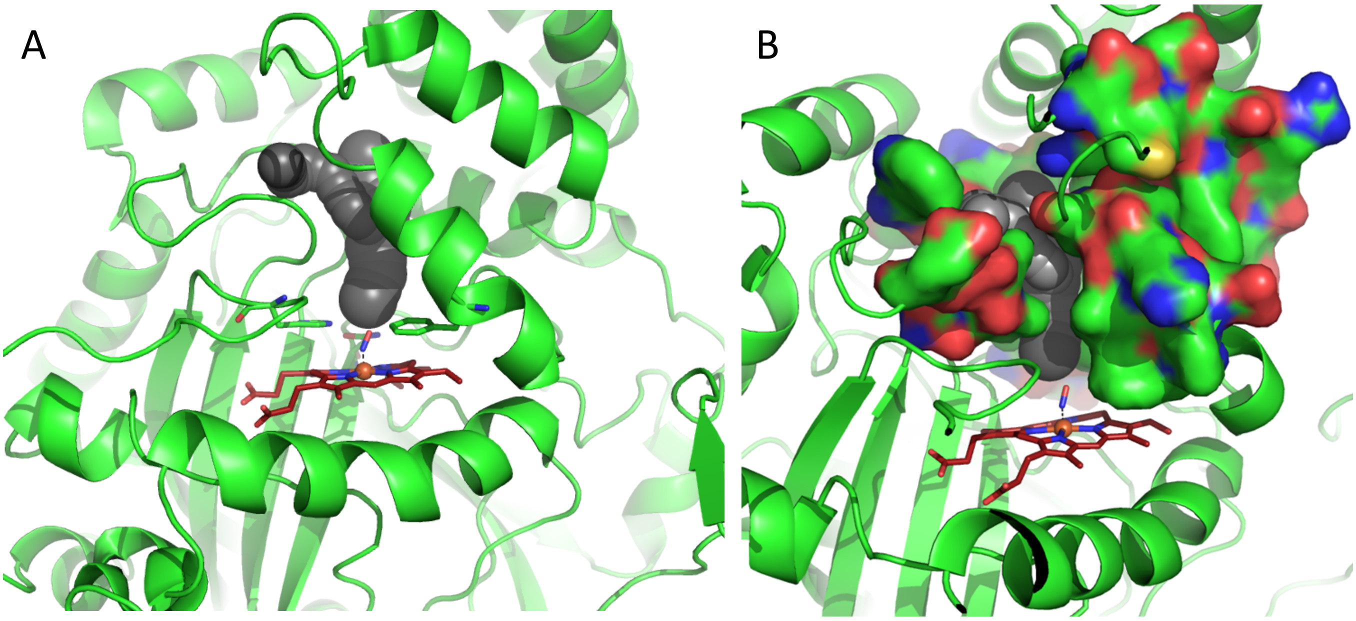

Figure S9: Modified view of Fig 5 from main text. Water accessible cavity (black surface) in the NO-bound Catalase (pdb: 4b7f).[1] The cavity volume, estimated with Caver,[2] corresponds to ~ 200 Å^3. Protein backbone is shown as a green cartoon in A and as green, solvent-excluded surface in B. Images are 30º apart from each other, rotated along the vertical axis. Coordination bonds are shown as dashed lines. Haem is shown as red sticks.

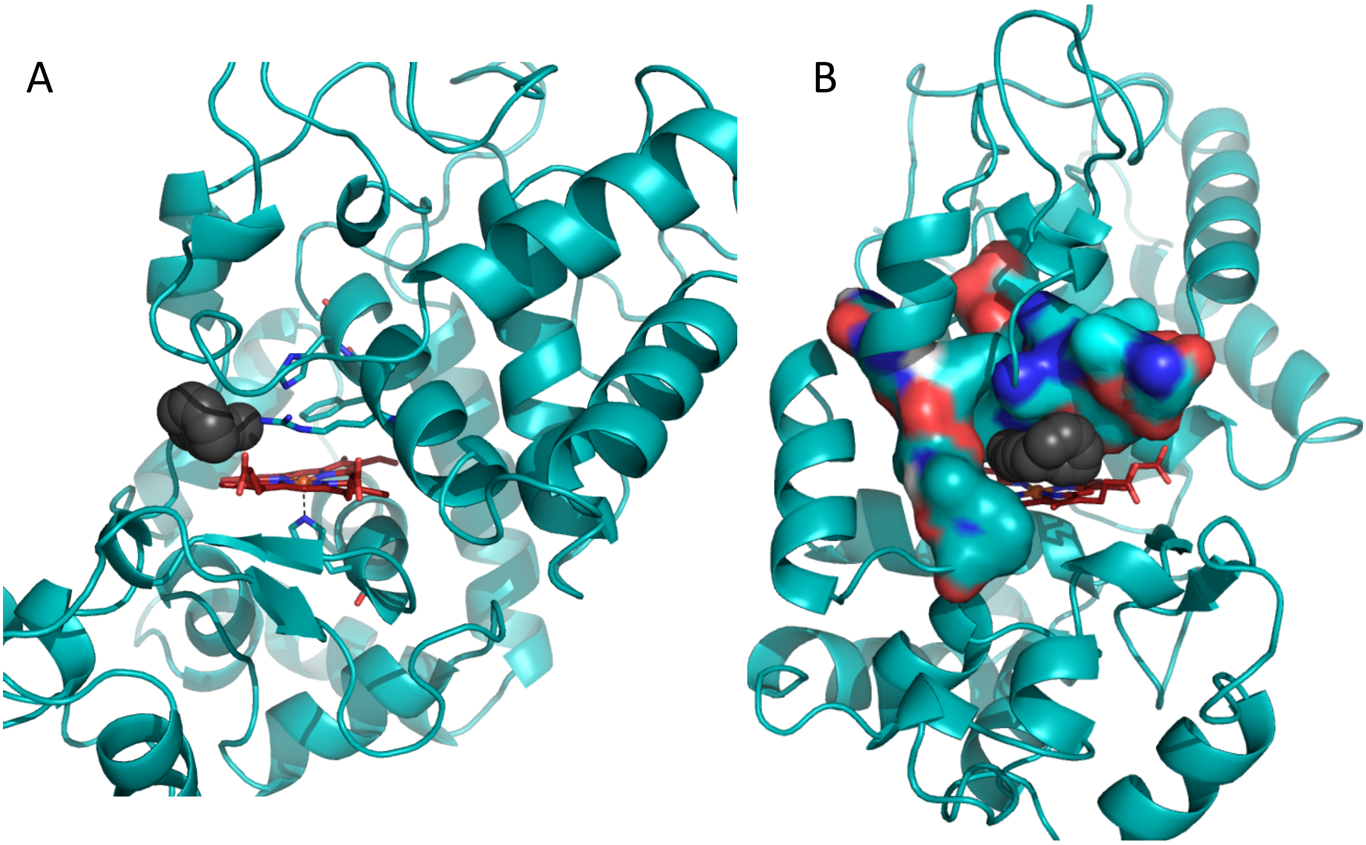

Figure S10: Water accessible cavity (black surface) in HRP (pdb: 1atj).[3] The cavity volume, estimated with Caver[1] corresponds to approx. 60 Å^3. The protein backbone is shown as a cyan cartoon in A and as cyan, solvent-excluded surface in B. Images are 90º apart from each other, rotated along the vertical axes. Coordination bonds are shown as dashed lines. Haem is shown as red sticks.

References

(1) Candelaresi, M.; Gumiero, A.; Adamczyk, K.; Robb, K.; Bellota-Antón, C.; Sangul, V.; Munnoch, J. T.; Greetham, G. M.; Towrie, M.; Hoskisson, P. A.; Parker, A. W.; Tucker, N. P.; Walsh, M. A.; Hunt, N. T. *Org Biomol Chem* **2013**, *11*, 7778.

(2) Kozlikova, B.; Sebestova, E.; Sustr, V.; Brezovsky, J.; Strnad, O.; Daniel, L.; Bednar, D.; Pavelka, A.; Manak, M.; Bezdeka, M.; Benes, P.; Kotry, M.; Gora, A.; Damborsky, J.; Sochor, J. *Bioinformatics* **2014**, *doi: 10.1093/bioinformatics/btu364*.

(3) Gajhede, M.; Schuller, D. J.; Henriksen, A.; Smith, A. T.; Poulos, T. L. *Nature Structural Biology* **1997**, *4*, 1032.
